# Characterisation of synovial fluid and infrapatellar fat pad derived mesenchymal stromal cells: The influence of tissue source and inflammatory stimulus

**DOI:** 10.1038/srep24295

**Published:** 2016-04-13

**Authors:** John Garcia, Karina Wright, Sally Roberts, Jan Herman Kuiper, Chas Mangham, James Richardson, Claire Mennan

**Affiliations:** 1 The Robert Jones and Agnes Hunt Orthopaedic Hospital NHS Foundation Trust, Oswestry, Shropshire, SY10 7AG, UK; 2 Institute for Science & Technology in Medicine, Keele University, Keele, Staffordshire, ST5 5BG, UK

## Abstract

The infrapatellar fat pad (FP) and synovial fluid (SF) in the knee serve as reservoirs of mesenchymal stromal cells (MSCs) with potential therapeutic benefit. We determined the influence of the donor on the phenotype of donor matched FP and SF derived MSCs and examined their immunogenic and immunomodulatory properties before and after stimulation with the pro-inflammatory cytokine interferon-gamma (IFN-γ). Both cell populations were positive for MSC markers CD73, CD90 and CD105, and displayed multipotency. FP-MSCs had a significantly faster proliferation rate than SF-MSCs. CD14 positivity was seen in both FP-MSCs and SF-MSCs, and was positively correlated to donor age but only for SF-MSCs. Neither cell population was positive for the co-stimulatory markers CD40, CD80 and CD86, but both demonstrated increased levels of human leukocyte antigen-DR (HLA-DR) following IFN-γ stimulation. HLA-DR production was positively correlated with donor age for FP-MSCs but not SF-MSCs. The immunomodulatory molecule, HLA-G, was constitutively produced by both cell populations, unlike indoleamine 2, 3-dioxygenase which was only produced following IFN-γ stimulation. FP and SF are accessible cell sources which could be utilised in the treatment of cartilage injuries, either by transplantation following *ex-vivo* expansion or endogenous targeting and mobilisation of cells close to the site of injury.

Regenerating joint tissues that have been damaged by trauma or degenerative diseases, such as osteoarthritis (OA), continues to present a challenge to scientists and clinicians world-wide. Autologous chondrocyte implantation (ACI) is a two-stage procedure that involves harvesting healthy cartilage arthroscopically in the first stage, followed by implantation of culture expanded cells at the damaged site via open surgery in the second stage, in an attempt to induce repair of the damaged tissue^1^. Eighty one percent of patients treated at our centre showed improved joint function and pain reduction (assessed via Lysholm scores) over an average follow-up time of 5 years^2^. However, ACI has some disadvantages relating to cost and possible donor site morbidity^3^,^4^,^5^, which limits its current use as a standard treatment for cartilage repair. Mesenchymal stromal cells (MSCs) have been isolated from many different tissue sources, including bone marrow (BM-MSCs), adipose tissue (commonly called adipose stem cells, ASCs), synovium (SM-MSCs) and umbilical cord (UC-MSCs) and are promising candidates for use in regenerative cell-based therapies. The infrapatellar fat pad (FP) and synovial fluid (SF) also represent accessible sources of MSCs (FP-MSCs and SF-MSCs, respectively) which are often routinely removed as surgical waste during arthroscopy or open knee surgery. The precise origin of SF-MSCs is not known; reports propose that they come from different tissues within the knee such as cartilage, bone and synovium, with synovium often considered as the most likely^6^,^7^,^8^. In an *in vitro* model, one study has illustrated that the joint SF of OA patients enhances MSC migration from synovium explants, a process believed to be mediated by transforming growth factor beta-3 (TGF-β3)^9^.

Better characterisation of these cells is vital, not only to ensure their safety for *in vivo* use, but also to test their therapeutic efficacy. However, a number of parameters such as tissue source and donor specificity are often overlooked by researchers, despite the clear evidence indicating that these features influence the phenotype of MSCs. It has been demonstrated that both donor age and gender affect the differentiation of BM-MSCs and A-MSCs; male and young donors generally produce MSCs with a more enhanced osteogenic and chondrogenic potency^10^,^11^,^12^. Further, older donors are known to produce MSCs that are less proliferative compared to younger donors^12^. These findings collectively suggest a clear influence of the donor on the phenotype of MSCs, and support the need for more robust studies that consider tissue origin and donor features as important parameters for the development of cell therapies.

In addition to the minimum characterisation criteria of MSCs established by the International Society for Cell Therapy (ISCT)^13^ which assesses cell surface markers and multipotent ability, understanding the immunological properties of MSCs in response to a pro-inflammatory stimulus has also been proposed as an important part of their characterisation^14^. BM-MSCs have been shown to increase their production of the immunomodulatory molecule indoleamine 2, 3-dioxygenase (IDO)^15^ in response to stimulation with the pro-inflammatory cytokine interferon-gamma (IFN-γ). IDO is an enzyme involved in the depletion of the essential amino acid, tryptophan, resulting in the inhibition of the proliferation of microbes and T cells^16^. Like IDO, the human leukocyte antigen-G (HLA-G) is another important immunomodulatory molecule which exerts its immunosuppressive effect on activated T cells^17^. It is produced by BM-MSC and by the trophoblast cells in the placenta where it plays a role in maternal tolerance to the foetus. The human leukocyte antigen-DR (HLA-DR), a major histocompatibility complex class II (MHCII) molecule, is found on antigen presenting cells (APCs) of the immune system and interacts with T cell receptors during an immune response. HLA-DR is not inherently expressed on BM-MSCs, but is known to be upregulated following a pro-inflammatory stimulus^18^. In contrast, the costimulatory markers CD40, CD80 and CD86, which are also produced by APCs involved in T cell mediated immune reactions^19^, are not produced on BM-MSCs even after inflammatory stimulation^20^. While FP-MSCs and SF-MSCs have been previously characterised in terms of their growth, multipotency and CD profile^7^,^21^, the immunological and immunomodulatory properties of these cells have not been thoroughly defined. The purpose of this investigation was to assess the influence of tissue source and donor on the properties of MSCs sourced from donor matched FP and SF isolated from human knees. We also investigated the previously unexplored response of FP- and SF-MSCs to a pro-inflammatory stimulus, such as that which may be found in an osteoarthritic joint.

## Results

### Macroscopic observations of FP, Histology, Cell culture and Growth kinetics

**Figure 1:**
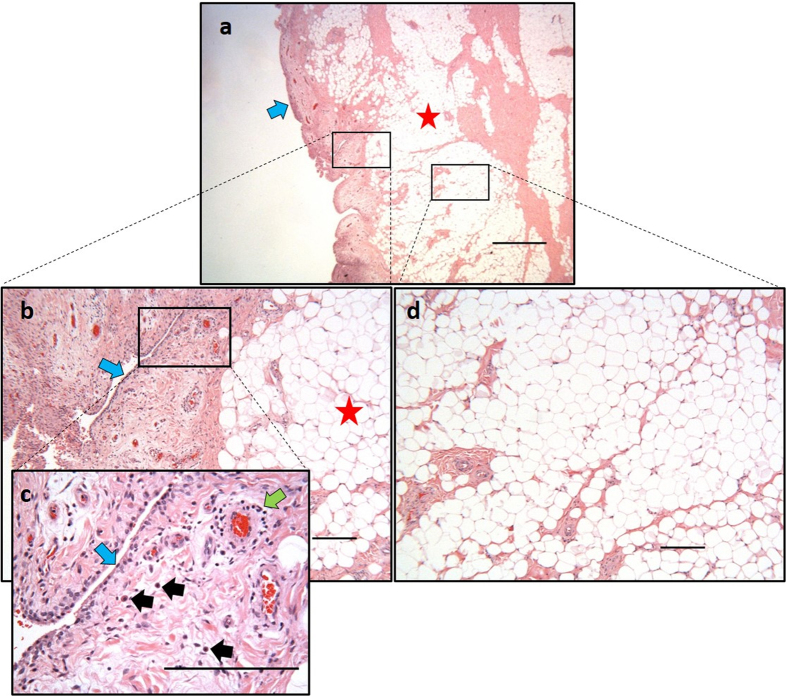
Example histology of a fat pad. Samples were paraffin embedded and stained with H&E. (**a)** Low power view of the fat pad as received after a knee replacement showing synovium (blue arrow) and adipose tissue (red star). (**b)** The deep adipose tissue is indicated by a red star and the adjacent synovium is indicated by a blue arrow. (**c)** High magnification view of the synovium with a layer of synoviocytes (blue arrow), mast cells (black arrows), and a blood vessel surrounded by a cuff of lymphocytes (green arrow). (**d)** Representative region of deep-lying vascularised adipose tissue from which FP-MSCs are isolated. Scale bars represent 1 mm (**a**) and 200 μm (**b–d**).

Donor variability was noted in the apparent level of inflammation observed by the surgeon (Table 1). The complex and heterogeneous features of the intact FP tissue were clearly visible in our histological preparations showing synovium and adipose regions (Fig. 1a). Blood vessels appeared scattered throughout the synovium (Fig. 1c) and deeper adipose tissue (Fig. 1b). For some samples a layer of synovial cells could be found on the superficial regions of the synovium along with mast cells, lymphocytes and numerous blood vessels (Fig. 1c). To avoid contamination of adipose derived cells with synoviocytes, only the deepest areas of the FP were used for digestion, as highlighted in Fig. 1d.

**Figure 2:**
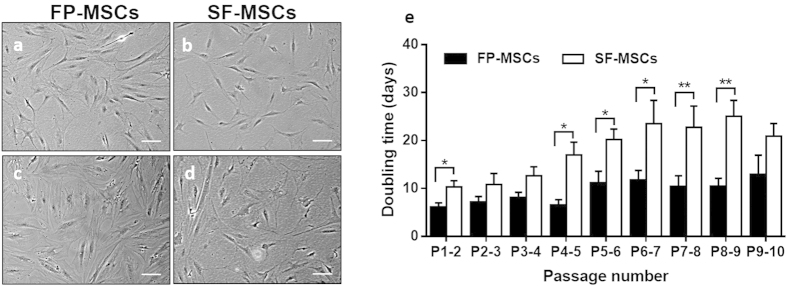
Microscopic photographs and growth kinetics of FP- and SF-MSCs. (**a–d)** Representative images were taken at passage 2 (**a,b**) and passage 10 (**c,d**). Scale bars represent 100 μm. (**e)** Growth kinetics data represents the mean doubling time ± standard error of the mean (SEM) for 6 donor-matched FP and SF samples. Over 10 passages, SF-MSCs show a significantly longer doubling time compared to FP-MSCs (P = 0.018). *P < 0.05, **P < 0.01.

MSCs isolated from both FP and SF were plastic adherent and appeared to consistently have a fibroblastic-like morphology at early passage (P2) through to the last passage tested (P10) (Fig. 2a,b). The results of the hierarchical regression analysis (Table 2) show that the average doubling time (DT) of SF-MSCs was 8.3 days longer than the DT of FP-MSCs (P < 0.001) over the 10 passages tested, for all donors. The DT increased with passage number by 1.4 days per passage and this increase was significantly dependant on the donor (P < 0.001).

### Immunoprofile of FP-MSCs and SF-MSCs

**Figure 3:**
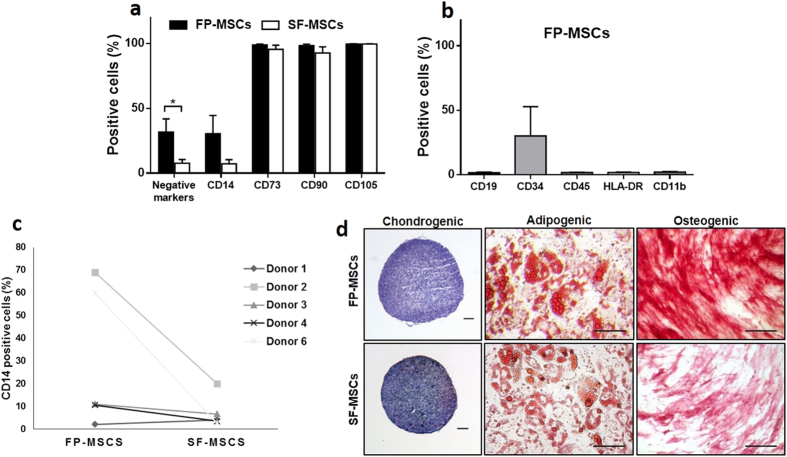
MSC immunoprofile (via flow cytometry) and trilineage differentiation of FP and SF-MSCs. (**a)** Both FP and SF-MSC populations were immunopositive for CD73 (98.8%), CD90 (98.3%), CD105 (99.6%); FP-MSCs showed significantly more cells which were positive for the cocktail of ISCT defined negative MSC selection markers compared to SF-MSCs (P = 0.046). (**b)** Single antibody cytometric analysis revealed a high positivity for CD34 in FP-MSCs (n = 3), but no positivity for CD11b, CD19, CD45 and HLA-DR. Error bars indicate SEM. (**c)** Cell positivity for CD14 was variable between donors and matched cell populations (n = 5). (**d)** The two MSC populations demonstrate differentiation potential when grown in different media; Chondrogenesis is shown by GAG production (purple metachromasia when stained with toluidine blue), adipogenesis is shown by the formation of lipid vesicles (oil red O) and osteogenesis is shown by the activity of alkaline phosphatase (red staining). Images are representative of the differentiation potential of all six donor matched samples. Scale bars represent 100 μm.

Flow cytometry analyses (Fig. 3a) revealed that both cell populations were over 95% positive for the MSC markers CD73 and CD105, but CD90 showed only 92.7% ± 11 positivity in SF-MSCs but 98.3% ± 3.1 in FP-MSCs. Interestingly, some positivity was recorded for the cocktail of PE conjugated markers expected to be negative on MSC cultures i.e. CD11b, CD19, CD45, CD34 and HLA-DR. FP-MSCs showed significantly (P = 0.046) greater positivity for the “negative” MSC markers (31.7% ± 24% of cells) compared to cells sourced from SF (7.8% ± 6.9% of cells). To explore this further, single PE-conjugated antibodies for CD11b, CD19, CD45, CD34 and HLA-DR were used to analyse FP-MSCs. FP-MSCs were negative for all of these markers, with the exception of CD34 which was 30.1% ± 18.6% positive (Fig. 3b). The macrophage lineage and inflammation marker CD14 was present on both FP-MSCs (30.5% ± 30.3%) and SF-MSCs cells (7.4% ± 7.2%, P = 0.14), with some variability observed between donors (Fig. 3c). An increase in CD14 positive was associated with donor age for SF-MSCs (n = 5, *r* = 0.94, P = 0.016), but not FP-MSCs (n = 5, *r* = 0.66, P = 0.23)

### Mesenchymal Trilineage Differential Potential

After 21 days of differentiation, all donor matched samples showed multipotent capability (Fig. 3d). FP-MSCs and SF-MSCs in adipogenic medium produced lipids which stained positively with oil red O, with those produced by FP-MSCs being more numerous and larger than those produced by SF-MSCs. Furthermore, they also showed stronger staining for alkaline phosphatase compared to SF-MSCs, which suggests greater osteogenic potential for the FP-MSCs. Histological staining for metachromasia using toluidine blue of chondrogenic cell pellets after differentiation revealed a similar level of GAG production by both cell populations, with no discernible difference in staining intensity between donors.

### Immunogenic and immunomodulatory properties of FP-MSCs and SF-MSCs after IFN-γ stimulation

**Figure 4:**
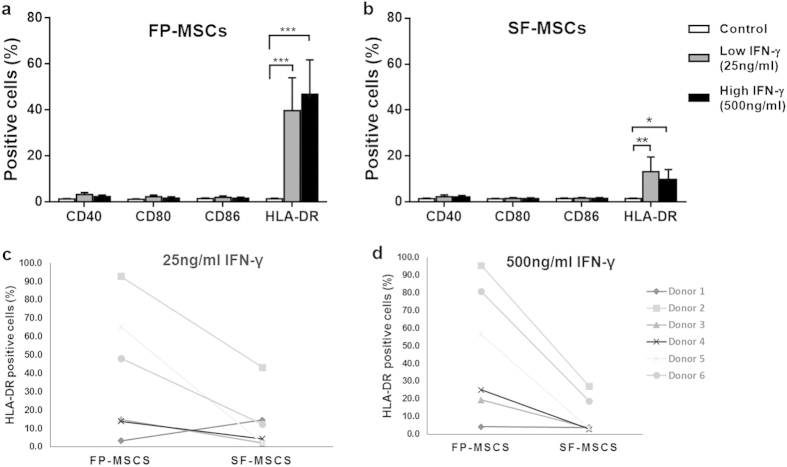
Immunogenic properties of FP and SF-MSCs after stimulation with IFN-γ (error bars indicate SEM). **(a)** The production of co-stimulatory markers (CD40, CD80, and CD86) and the class II MHC receptor HLA-DR for FP-MSCs is shown after 48 hours of stimulation with IFN-γ along with unstimulated controls. FP-MSCs do not produce co-stimulatory molecules regardless of stimulation, but show greater HLA-DR positivity at 25 ng/ml (39.8% ± 14.3, *p* < 0.0001) and at 500 ng/mL (46.9% ± 14.9, *p* < 0.0001) stimulation with IFN-γ. (**b)** SF-MSCs show no positivity for co-stimulatory marker, even when stimulated, but produce significantly higher levels of HLA-DR at 25 ng/ml (13.1% ± 6.4, P = 0.0015) and at 500 ng/mL IFN-γ (9.8% ± 4.3, P = 0.025). (**c–d)** Donor variability in the levels of induced HLA-DR on FP-MSCs and SF-MSCs was observed at both 25 ng/mL (C) and 500 ng/ml (D). However, donor number 2 (indicated by the square data points) had higher positivity for HLA-DR compared to other donors for both FP-MSCs and SF-MSCs and for both of the IFN-γ doses tested.

After 48 h of treatment with the either 25 or 500 ng/ml of the pro-inflammatory cytokine, IFN-γ, analysis via flow cytometry showed that CD40, CD80 and CD86 were not present on FP-MSCs or SF-MSCs and were similar to the untreated controls (Fig. 4a,b). In contrast, a low concentration (25 ng/ml) of IFN-γ significantly increased the positivity for HLA-DR in both cell populations compared to their respective controls (FP-MSCs = 39.8% ± 35.1%, SF-MSCs = 13.1% ± 11.7%). FP-MSCs also showed a significantly higher percentage of HLA-DR positive cells (46.9% ± 36.5) compared to SF-MSCs (10.0% ± 10.4) at 500 ng/ml IFN-γ (P = 0.04). Donor variability was noted with regards to the induction of HLA-DR by IFN-γ, however MSCs derived from both the FP and SF of donor number 2 consistently produced more HLA-DR in response to IFN-γ stimulation compared to the other donors, at both doses tested (Fig. 4c,d). After stimulation with 25 ng/ml of IFN-γ, FP-MSCs showed more production for HLA-DR, which was positively correlated with an increase in donor age (n = 6, r = 0.82, P = 0.045); SF-MSCs showed no such trend (n = 6, *r* = 0.72, P = 0.105).

**Figure 5:**
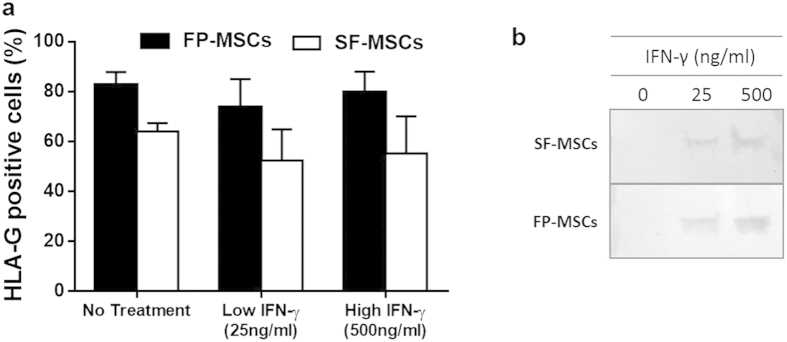
Immunomodulatory properties of FP and SF-MSCs after stimulation with IFN-γ (error bars indicate SEM). **(a)** Both FP and SF-MSCs (n = 3) inherently produce HLA-G in control conditions (82.7% ± 5.1 and 64.0% ± 3.4 respectively), as well as following treatment with 25 ng/ml (73.8% ± 11.2 and 52.4% ± 12.5 respectively) and 500 ng/ml IFN-γ (79.7% ± 8.3 and 5.2% ± 14.9 respectively). (**b)** Cropped image of a representative western blot indicating the production of IDO by FP-MSCs and SF-MSCs after stimulation with IFN-γ, but not in untreated controls, a full-length blot is presented in Supplementary Figure S1.

Both cell populations produced the immunomodulatory marker, HLA-G, regardless of inflammatory stimulus and with no significant difference observed between FP-MSCs and SF-MSCs (Fig. 5a). Western blots showed that the production of IDO in both cell populations was induced by IFN-γ stimulation but was absent in unstimulated controls (Fig. 5b).

## Discussion

In this study, we have characterised the endogenous population of MSCs found in patient matched samples of infrapatellar FP and joint SF, and explored the possible influence of a pro-inflammatory stimulus on the immunogenic and immunomodulatory properties of these cells. Histology of the FP and synovium revealed the presence of immune cells, notably mast cells and lymphocytes, in the superficial regions of the tissues. Both of these cell types are known to be present in synovial tissues of arthritic patients and produce molecules that promote joint inflammation^22^,^23^,^24^. Macroscopic inspection of the FP during surgery in the present study revealed some degree of inflammation as indicated by the size of the FP itself and redness of the attached synovium. Our results show that both FP- and SF-MSCs are plastic adherent cells with similar fibroblast-like morphologies *in vitro*. We have noted a significant increase in the doubling times of SF-MSCs at late passages (compared to FP-MSCs), which suggests that SF-MSCs lose proliferative capacity earlier than FP-MSCs. Reports have indicated that both human BM-MSC and ASC doubling times increase with passage number^25^,^26^,^27^; a longer culture period may have been required in the current study in order to observe a similar reduction in FP-MSC proliferation. The consistent difference in growth rate observed between FP-MSCs and SF-MSCs, isolated from matched donors, highlights that these cells are biologically different. Interestingly, an investigation observing patient-matched SM-MSCs and SF-MSCs revealed no significant difference in the proliferation rate of the two cell types between P0 and P2^28^, perhaps providing supportive evidence that these cells share a common origin.

In agreement with previous studies^6^,^29^, we confirm the multipotency of both cell populations, with FP-MSCs showing enhanced differentiation towards adipogenic and osteogenic lineages compared to SF-MSCs. Also, as previously described^7^,^29^ we have demonstrated that both FP-MSCs and SF-MSCs populations possess chondrogenic ability, but we have also shown that there was no discernible difference in the chondrogenic potential of FP-MSCs or SF-MSCs derived from the same donor. Our results show that these donor-matched SF-MSCs and FP-MSCs are positive for MSC cell surface markers CD73, CD90, CD105, as well as many of them being positive for the myeloid lineage marker CD14^30^. This observation indicates that joint SF and the stromal fraction of FP contain cells that in part meet the ISCT criteria for MSCs, but may also harbour a sub-population that do not i.e. those with CD14 positivity. CD14 positivity has previously been reported on BM-MSCs^31^, but not FP-MSCs or SF-MSCs to date. Macrophage-like synoviocytes, which are also positive for CD14, are a likely contaminant sub-population contributing to the heterogeneity of our cultures^32^,^33^. SF is known to contain CD14 positive macrophages that secrete pro-inflammatory cytokines, such as IL-1 and TNF-α, that induce enzyme-mediated cartilage degradation^34^,^35^. Furthermore, there is increased detectable soluble CD14 in the SF of osteoarthritic patients^36^. Hence, the positive association of CD14 in SF-MSCs and advancing age could be indicative of an increase in pro-inflammatory conditions in the degenerative joints of older subjects^37^,^38^,^39^.

No single surface marker that we investigated allowed a clear distinction between FP-MSCs and SF-MSCs, which suggests that a broader panel of markers would be required in order to distinguish between these two cell types. Recent guidelines from the ISCT and the International Federation for Adipose Therapeutics and Science now accept CD34 as being positive on adipose stromal cells^40^. CD34 positive cells in FP-MSCs and other ASCs cultures^41^,^42^, are likely to represent subpopulations of either vascular endothelial lineages, pericytes or a mixture of the two, as found in cells isolated from subcutaneous lipoaspirate^34^. Further work would be required to isolate and phenotypically characterise these CD34 positive cells within adipose tissues to assess their function in native and transplanted tissues.

In addition to surface antigens and differentiation characterisation criteria, the need to understand the immunological properties of MSCs is becoming crucial to the development of cell therapies^14^,^43^, particularly in the treatment of conditions which involve inflammatory elements as is the case for OA^44^,^45^. APCs interact with T cells via MHC class II molecules, such as HLA-DR, to trigger an alloresponse^46^. This reaction involves the activation, differentiation and proliferation of T cells but is not possible without the expression of costimulatory surface molecules^47^. The results we present here are the first to show an increased positivity of MHC class II and a lack of expression of costimulatory molecules on matched FP-MSCs and SF-MSCs in response to an *in vitro* inflammatory stimulus. BM-MSCs stimulated with IFN- γ have been shown to produce levels of HLA-DR that are comparable to our observations with FP-MSCs^20^. Conversely, the immunoprofile of IFN-γ induced HLA-DR on SF-MSCs was much lower, perhaps indicating that SF-MSCs are less immunogenic following inflammatory stimulation and hence display a distinct immunological phenotype compared to FP-MSCs and also BM-MSCs. This is the first time, to our knowledge, that a difference in positivity for HLA-DR on stimulated MSCs derived from the same joint has been reported and requires further investigation. Inflammation of the knee, due to elevated levels of pro-inflammatory molecules, has been reported to increase with age^39^,^48^. Our results indicate an increasing trend with age in the production of HLA-DR by FP-MSCs after exposure to IFN-γ. It could be hypothesised that the inflammatory nature of the FP is increased with age and in turn pre-conditions the endogenous MSC niche to respond to IFN-γ by augmenting HLA-DR positivity. The reason why this correlation was not reproduced in the SF-MSCs derived from the same joints remains undetermined, but this observation highlights another biological difference between the two cell populations. *In vivo* investigations evaluating the general effects of natural aging and inflammation on MSC pools within the human knee joint could not only elucidate the cellular pathways involved in the pathogenesis of chronic joint diseases, but may also reveal potential therapeutic targets (genetic, molecular or cellular) to counter the degenerative processes. Despite the presence of HLA-DR on FP- and SF-MSCs in this study, these cells cannot be considered as truly immunogenic without an accompanying increase in the production of costimulatory markers^47^,^49^.

It is perhaps also noteworthy that the only donor included in this study observed in surgery to have a ‘severely inflamed’ infrapatellar fat pad (donor 2) produced FP-MSCs and SF-MSCs which showed the most pronounced response to an inflammatory stimulus. We suggest that cells derived from such severely inflamed joints are ‘primed’ to be more responsive to inflammatory stimuli, but we appreciate that a larger cohort of donors with better defined (macroscopic and histologically quantified) tissues would be required to confirm this hypothesis.

The immunosuppressive properties of SF-MSCs have been demonstrated in mixed lymphocyte reactions^50^, but the constitutive production of HLA-G in FP-MSCs and SF-MSCs has not been reported previously. A similar level of HLA-G production has, however, been observed in both BM-MSCs and UC-MSCs^17^,^20^. HLA-G positive UC-MSCs have been shown to inhibit the proliferation of pro-inflammatory T helper 1 cells, which are present in OA joints^51^, and promote the expansion of anti-inflammatory regulatory T cells. Further, we have demonstrated that the production of IDO can be induced in both cell populations by an inflammatory stimulus, comparable to BM-MSC and ASC cultures^52^,^53^. In a collagen induced arthritis mouse model, IDO deficiency was associated with a high incidence of arthritis and pronounced T cell infiltration^54^. The pro-inflammatory conditions within the OA joint could potentially activate the local MSC populations to dampen the influence of certain immune cells such as T cells and macrophages. The present study suggests that FP-MSCs and SF-MSCs may be considered as immunomodulatory cells but they would require more in depth *in vitro* functional testing alongside BM-MSCs to fully assess their therapeutic value in this respect. Others have shown that conditioned media derived from BM-MSCs that were stimulated with IFN-γ and TNF-α, reduced the gene expression of pro-inflammatory and cartilage degrading molecules in synovial and cartilage explants *in vitro*^55^. This trophic feature remains largely undefined in FP- and SF-MSCs but may provide insight into the possible mechanism of action that these cells might have in an arthritic joint.

The use of donor matched samples in our investigations has allowed us to better identify and analyse donor-specific variability. Tissue source appears to be more influential on the proliferative and differentiation properties of joint derived MSCs than the donors themselves or related factors. The reasons for this are still unclear. An increase in donor age, for example, is known to negatively affect the differentiation potential and growth rate of BM-MSCs^56^ and subcutaneous ASCs^12^,but this does not seem to apply for the FP- and SF-MSCs observed in our study.

Donors included in this study were predominantly patients with chondral defects and with early signs of OA. However, one donor was described as having end stage OA and was thus undergoing total joint replacement. This heterogeneity in samples may account for some of the variability observed, but also provides insight into the inherent biological differences in donors that should be considered in the development of new cartilage repair strategies.

## Conclusion

We have characterised the phenotype and have identified biological distinctions between MSCs sourced from the FP and SF within the same articular joint. We have confirmed that there is obvious potential for the use of FP-MSCs or SF-MSCs in the treatment of cartilage defects following *ex-vivo* expansion. However, it may be more advantageous to find means of recruiting these resident MSCs with chondrogenic potency from their respective niches *in vivo*, as opposed to current methods of transplanting expanded cells. The advantage of this approach would be that the significant costs and regulatory hurdles involved with the *in vitro* manufacturing of cells may be circumvented.

Further, we have shown that the levels of CD14 on SF-MSCs and the inducibility of HLA-DR on FP-MSCs are possibly due to age-related inflammation. We confirm for the first time, an immunomodulatory capacity for FP- and SF-MSCs which confers further therapeutic value to these cells with regards to treating the inflammatory aspect of OA. This also highlights the need to understand the physiological inflammatory conditions into which transplanted cells are place and how this could affect the outcome of a cellular treatment. FP-MSCs and SF-MSCs may not realistically be suited for large-scale allogeneic therapies due to limited sample availability and low cell numbers, but certainly represent sources of autologous cells for cartilage regeneration in addition to sourcing chondrocytes from healthy cartilage, as is used currently. Further investigations will seek to understand how these MSCs can be directed to effect repair endogenously either by means of dampening down inflammatory processes or by producing repair cartilage themselves or even by encouraging reparative cellular recruitment.

## Methods

### Isolation and culture of FP-MSCs and SF-MSCs

All samples were obtained after patients had provided written informed consent; favourable ethical approval was given by the National Research Ethics Service (11/NW/0875) and all experiments were performed in accordance with relevant guidelines and regulations. FP and joint SF were obtained from 6 patients (3 males and 3 females, ages 35–79 years) undergoing either total knee replacement or ACI surgery. At the time of surgery a macroscopic observation of inflammatory state of the FP was made by the operating surgeon and is shown in Table 1, along with all other donor demographics. The FP was dissected from the deepest zone, to avoid contamination with synovium derived cells. To confirm clear dissection of the two tissues, portions of selected samples were fixed and processed for paraffin wax embedding and routine histological study via haematoxylin and eosin staining. FP tissue was washed in phosphate buffered saline (PBS, Life Technologies Paisley, UK), minced into 2–3 mm^3^ pieces and digested with 1 mg/ml collagenase type I (>125 digesting units/mg, Sigma-Aldrich, Poole, UK) in Dulbecco’s Modified Eagle’s Medium/F-12 (DMEM/F-12, Life Technologies) with 1% (v/v) penicillin/streptomycin (P/S) (Life Technologies) for 1 h at 37 °C. The resulting cells were strained through a 40 μm nylon cell strainer and centrifuged (350*g* for 10 min). Cells were then seeded at a density of 5000 cells/cm^2^ in the above media supplemented with 10% (v/v) foetal calf serum (Life Technologies). FP was prepared for histology via paraffin embedding and sectioned at 5 μm using a Shandon SME cryotome (Thermo Scientific Loughborough, UK) and tissue sections were collected onto adhesive slides (Leica Biosystems Surgipath, Nussloch, Germany). Haematoxylin and eosin (H and E) staining was conducted as per standard procedure.

SF was centrifuged (800*g* for 15 minutes) and the resulting pellet seeded into a T25cm^2^ culture flask. All cultures were maintained at 37 °C and 5% CO_2_ in a humidified atmosphere. Media was replaced every 2–3 days. At 70% confluence, growth media was removed and the cells washed with PBS to remove residual serum and media. Cells were then detached with 0.05% (v/v) trypsin-EDTA (Life Technologies) and reseeded at 5000 cells/cm^2^.

### Growth Kinetics

The proliferation rate of the cells was evaluated by calculating their DT over ten passages (P). Cells were seeded into T25 cm^2^ flasks at a density of 5000 cells/cm^2^ and allowed to proliferate to 70–80% confluence before trypsinisation and reseeding. The cell population DT was calculated using the following equation:


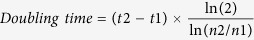


where t2-t1 is the number of days in culture, n2 is the number of cells recovered after passage and n1 is the total number of cells seeded.

### Microscopy

Histological sections were viewed by light microscopy (Leitz, Wetzlar, Leica Microsystems GmbH, Germany) using ×6.3, ×25 and ×40 objective lenses and a DS-Fi1 camera (Nikon Corporation, Japan), images were analysed using NIS-Elements BR software version 3.2 (Nikon). Cells in monolayer culture were digitally imaged by phase contrast microscopy using a ×10 objective lens (Nikon Eclipse TS100) and a C4742-9S camera (Hamamatsu, Japan), images were analysed using IPLab v3 software (BD Biosciences).

### Flow cytometry Immunoprofiling

Flow cytometry was used to assess the immunoprofile of MSCs derived from FP and SF. Cells at P3–4 were harvested, pelleted, and re-suspended in 2% bovine serum albumin (BSA, Sigma-Aldrich, Poole, UK) in PBS. FC receptors were blocked for 1 h at 4 °C using 10% (v/v) human IgG (Grifols, UK) with 2% BSA in PBS. The cells were then washed with 2% (v/v) BSA in PBS and centrifuged (350*g* for 8 minutes). Cells were stained for (30 minutes at 4 °C) with fluorochrome conjugated antibodies against CD90-phycoerythrin (PE) (clone 5E10), CD105-allophycocyanin (APC) (clone 266), and CD73-brilliant violet 421 (BV421) (clone AD2) (BD Biosciences, Oxford, UK). A commercially available pre-mixed cocktail of PE-conjugated antibodies was initially used for the detection of markers CD11b (clone ICRF44), CD19 (clone HIB19), CD34 (clone 581), CD45 (clone HI30) and HLA-DR (clone TU36) (BD Biosciences) on FP-MSCs and SF-MSCs. Further staining for individual markers was performed for FP-MSCs with fluorochrome conjugated antibodies against CD11b-PE, CD19-BV421, CD34-APC, CD45-PE and HLA-DR-APC (clones stated previously) (BD Biosciences). The expression of CD14 was assessed using a peridinin chlorophyll protein-cyanine5.5 (PercP-Cy5.5) conjugated antibody (clone MϕP9) (BD Biosciences). Appropriate isotype-matched IgG controls were used throughout (BD Biosciences). Data from 10,000 stained cells is presented which was collected using a FACSCanto II flow cytometer (BD Biosciences) and analysed using BD FACSDiva v.7.0.

### Differentiation assays

The multipotency of FP-MSCs and SF-MSCs at P3 was assessed using osteogenic, adipogenic and chondrogenic differentiation protocols. The osteogenic, and adipogenic differentiation of FP-MSCs and SF-MSCs was investigated using monolayer cultures. Cells were seeded at a density of 5 × 10^3^/cm^2^ in 24 well plates (Sarstedt, Nümbrecht, Germany), and fed the appropriate differentiation medium, either osteogenic or adipogenic medium for 21 days. Osteogenic differentiation medium contained DMEM F12, FCS (10%), β-glycerophosphate (10 mM), dexamethasone (10 nM) and L-ascorbic-acid (50 μM). Adipogenic differentiation medium contained DMEM F12, FCS (10%), Insulin-transferrin-selenium-X (ITS) (1%) (Gibco UK), isobutylmethylxanthine (0.5 μM) (Sigma, UK), dexamethasone (1 μM) and indomethacin (100 μM). For control cultures, media containing DMEM F12, FCS (10%) and P/S was used. To evaluate the differentiation, cells were fixed with buffered formalin and stained with oil red-O for 1 h to assess lipid formation for adipogenesis or napthol-AS-BI phosphate and fast red for 1 h to assess alkaline phosphatase activity for osteogenesis.

A pellet culture system (2 × 10^5^ cells/pellet) was used to assess chondrogenic differentiation potential. Cells (2 × 10^5^) were centrifuged in sterile 1.5 ml microcentrifuge tubes with DMEM F12, FBS (10% v/v), P/S (1%) ITS (1%, v/v), ascorbic-acid (0.1 mM) (Sigma-Aldrich), dexamethasone (10nM) and transforming growth factor β-1 (TGF-β1, PeproTech, London, UK) (10 ng/ml)^57^. After 21 days, cell pellets were snap frozen in liquid nitrogen and stored at −80 °C prior to use. Pellets were cryosectioned (7 μM) onto poly-L-lysine coated slides (Cell Path, Newtown, UK) and stained for glycosaminoglycans (GAG) with toluidine blue (BDH) metachromatic stain. Sections were stained with toluidine blue for 30 seconds and then washed briefly in tap water. Slides were left to air dry before mounting in Pertex (Cell Path).

### Interferon-γ stimulation

The immunogenic nature of FP-MSCs and SF-MSCs was investigated by assessing the presence of the costimulatory markers CD40, CD80, CD86, and the class II Major Histocompatibility Complex (MHC), HLA-DR, after stimulation with the pro-inflammatory cytokine IFN-γ (20 units/ng, PeproTech). Cells at P3 were treated with culture medium containing either 25 ng/ml (low concentration) or 500 ng/ml (high concentration) of IFN-γ for 48 h. Untreated cells grown in standard culture medium were used as controls for comparison. After 48 h, cells were prepared for flow cytometry as described previously with PE-conjugated antibodies against CD40, CD80, CD86 (BD Biosciences) and HLA-DR (Immunotools, Friesoythe, Germany). HLA-G was assessed via flow cytometry using antibodies against surface and intracellular antigens (Santa-cruz, Texas, USA), using a Cytofix/CytopermTM and Fixation/Permeabilisation Kit (BD Biosciences) to the manufacturer’s recommendations.

### Western blotting for IDO detection

The production of IDO in cells at P3 was evaluated before and after stimulation with IFN-γ using western blotting. Cells were lysed with cold lysis buffer (100 μl lysis buffer per 1 × 10^6^ cells) containing 0.005% Tween 20, 0.5% Triton X-100 and protease inhibitors (Sigma-Aldrich, UK) at 4 °C, and kept at −20 °C until further use. After quantification of total protein using the bicinchoninic acid (BCA) assay (Life Technologies), 20 μg of each sample was loaded into pre-cast NuPAGE® 4–12% Bis-Tris gels (Life Technologies). An iBlot and transfer stacks system (Life Technologies) was used to transfer proteins onto nitrocellulose membranes. Primary and secondary antibodies employed to detect IDO were used at dilutions of 1:250 and 1:125, respectively, in antibody diluent. The primary antibody (Abcam, Cambridge, UK), against IDO, was applied first to the membrane, then the secondary antibody (1:125, Life Technologies) which was conjugated to horseradish peroxidase. Membranes were washed three times with a wash solution (Life Technologies), then twice with autoclaved water. A chromogenic substrate solution, NovexR Alkaline Phosphatase (Life Technologies), was added to the membranes, after which colour development was allowed for no longer than an hour. Membranes were washed again in autoclaved water before being allowed to dry in the dark prior to imaging.

### Statistical analyses

Statistical analyses were performed using GraphPad Prism (GraphPad Software, California, USA) and R (The R Foundation, Vienna, Austria). The Shapiro–Wilk test was used to assess the normal distribution of numerical data. The mean doubling times of FP-MSCs and SF-MSCs were compared using a two-way ANOVA with Bonferroni’s multiple comparisons tests, while a hierarchical regression analysis was used to determine the effect of donor, cell source and passage number on DT. A paired Student’s *t*-test was used to compare means and a Pearson’s test was used to establish correlations where appropriate. Graphs are shown as means ± standard error of the mean (SEM). Statistical significance was considered at *p < 0.05, ** *p* < 0.01 and ***p < 0.001.

## Additional Information

**How to cite this article**: Garcia, J. *et al.* Characterisation of synovial fluid and infrapatellar fat pad derived mesenchymal stromal cells: The influence of tissue source and inflammatory stimulus. *Sci. Rep.* **6**, 24295; doi: 10.1038/srep24295 (2016).

## Supplementary Material

Supplementary Information

## Figures and Tables

**Table 1:** Demographics of donors from which samples were sourced.

ID	Gender	Age (years)	Condition	Macroscopic observation of FP	Procedure
Donor 1	Male	35	Chondral defect on lateral femoral condyle	Mild inflammation	ACI
Donor 2	Female	42	Chondral defect on patella	Severe inflammation	ACI
Donor 3	Female	49	Osteochondral defect on patella	Hypertrophy	ACI
Donor 4	Female	49	Chondral defect on patella	Hypertrophy	ACI
Donor 5	Male	53	OA degeneration of knee with chondral defect on trochlea	General inflammation	ACI
Donor 6	Male	79	End-stage OA	General inflammation	Total knee replacement

**Table 2:** Results of the hierarchical regression analysis for the influence of cell source, passage number and donor on doubling time of matched FP- and SF-MSCs.

Factor	Fixed/Random effect	Coefficient or SD (days)	p-value
Cell source (SF)	Fixed	8.3 (6.1–10.4)	<0.001
Passage number	Fixed	1.4 (0.8–2.0)	<0.001
Passage by donor	Random	0.54 (0.19–1.1)	0.004

Note: Coefficients and standard deviation (SD) based on a linear mixed effect model. P-values are from Wald tests (fixed effects) and likelihood ratio tests (random effects).
