# Supplementary material for: Characterisation of synovial fluid and infrapatellar fat pad derived mesenchymal stromal cells: The influence of tissue source and inflammatory stimulus
Source: Sci Rep. 2016 Apr 13;6:24295. doi: 10.1038/srep24295 (PMC4829842; doi:10.1038/srep24295)
Supplement: Supplementary Information [file srep24295-s1.pdf]

Characterisation of synovial fluid and infrapatellar fat pad derived mesenchymal stromal cells: The influence of tissue source and inflammatory stimulus.

Authors: John Garcia, Karina Wright, Sally Roberts, Jan Herman Kuiper, Chas Mangham, James Richardson, Claire Mennan.

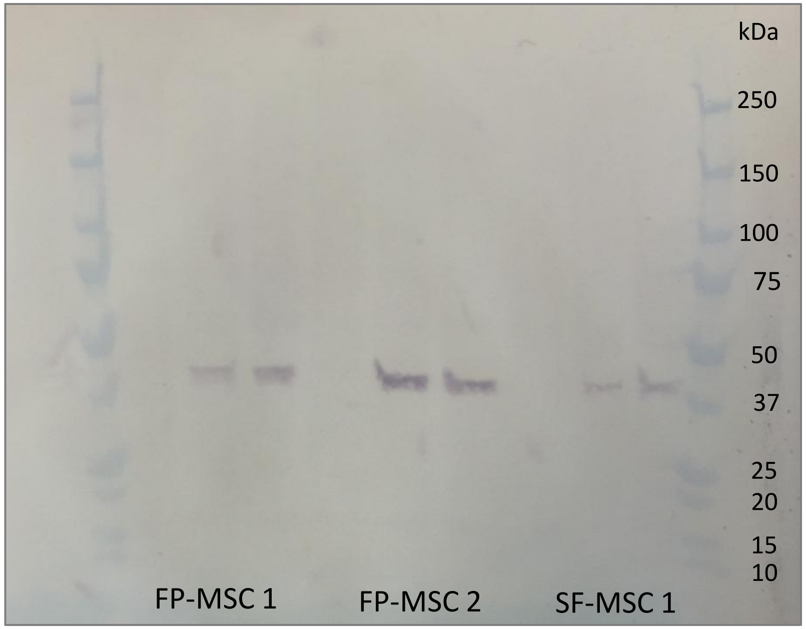

Supplementary Figure S1. Full Western blot image indicating the production of IDO (42 kDa) by FP-MSCs (FP-MSC 1) and SF-MSCs (SF-MSC 1) after stimulation with IFN-γ (25ng/ml and 500 ng/ml), but not in untreated controls.
